# A study protocol of a single-center investigator-blinded randomized parallel group study to investigate the effect of an acclimatization visit on children's behavior during inhalational sedation in a United Arab Emirates pediatric dentistry postgraduate setting as measured by the levels of salivary Alpha Amylase and Cortisol

**DOI:** 10.1097/MD.0000000000016978

**Published:** 2019-08-30

**Authors:** Manal Al Halabi, Iyad Hussein, Anas Salami, Rawan Awad, Najla Alderei, Ahtiq Wahab, Mawlood Kowash

**Affiliations:** a Hamdan Bin Mohammed College of Dental Medicine; b College of Medicine, Mohammed Bin Rashid University of Medicine and Health Sciences, Dubai, United Arab Emirates.

**Keywords:** acclimatization visit, anxiety, behavior, dental treatment, inhalation sedation, salivary amylase, salivary cortisol

## Abstract

**Introduction:**

Inhalation sedation is a proven safe method for reducing children's dental anxiety and has been used worldwide for decades. There is controversy regarding the use of acclimatization or familiarization visits for dental sedation treatment pathways for children. This may increase acceptance to the treatment based on desensitization and acclimatization principles underpinning many behavior management techniques. This study aims to identify whether, an inhalation sedation acclimatization visit is effective in reducing the stress level in anxious children as measured by salivary Alpha Amylase and Cortisol levels.

**Methods:**

The study is a single-center, single blinded, parallel group 2 arm clinical trial. Children in need of inhalation sedation aged 5 to 15 years from September 2019 through March 2020 attending the Postgraduate Pediatric Clinic at the Hamdan Bin Mohammed College of Dental Medicine, in Dubai, will be allocated randomly and equally to either:

(1) Study group: children and parents would attend a visit for prevention and inhalation sedation will be introduced and tried;
(2) Control group: children and parents would attend for prevention visit and discussion of inhalation sedation only.

At the initial visit a salivary sample will be collected at the beginning of the visit and the sedation need score will be recorded using the pediatric indicator of sedation need. Treatment for both groups will commence at the second visit. Salivary samples will be collected 15 minutes before the start of the treatment and 15 minutes after the conclusion of the treatment. The following outcomes will be recorded: completion of dental treatment, anxiety scores at baseline and after treatment using Frankl rating behavior scale and physiological anxiety related changes will be recorded using salivary Alpha Amylase and Cortisol levels. Mean changes of physiologic anxiety levels and corresponding 95% confidence intervals will be determined to compare the 2 treatments (sedation with familiarization and sedation without familiarization).

**Discussion:**

This is will be the first study to measure the effect of the acclimatization visit of nitrous oxide inhalation sedation on the level of physiological anxiety and the behavior of the pediatric patients during treatment.

## Abstract 2

Supplemental Digital Content is available in the text

## 1 Introduction

Inhalation sedation (IHS) is a light form of sedation. It is a mixture of nitrous oxide and oxygen breathed through a nosepiece. This helps the child to feel relaxed and accept treatment.^[1,2]^ It is well-known that dental fear and anxiety are most common barriers to seeking dental treatment. It has been reported that approximately 23 million people with dental fear would be more willing to visit a dentist if they have been offered a form of sedation.^[3]^

The use of IHS or general anesthesia (GA) is recommended to facilitate dental treatment when nonpharmacological behavior management techniques fail to alleviate children's anxiety and fear,^[2]^ especially in the United Arab Emirates (UAE), a country with high prevalence of dental caries.^[4,5]^

The administration of nitrous oxide IHS for dental procedures is highly dependent on the child's acceptance of and keeping the nitrous oxide nosepiece in place as, at the levels used for dental procedures, it only provides a mild level of sedation and anxiolysis.^[1,2]^

It is postulated that the use of an acclimatization (familiarization) visit which can be defined as one in which sedation is only provided and no, or minimal dental intervention, is carried out would increase the acceptance and hence the efficacy of the nitrous oxide IHS.^[2]^ This method can be controversial as on one hand it may increase acceptance based on desensitization and acclimatization principles underpinning many behavior management techniques. On the other hand, it exposes the child to an additional pharmaceutical intervention and increases contact time which may influence patient compliance.

The use of an acclimatization visit has been suggested in the literature. For example, Andlaw (1996) recommended the use of an acclimatization visit before dental treatment under nitrous oxide IHS.^[6]^ The United Kingdom Royal Colleges of Surgeons and the Royal College of Anaesthetists in 2015 recommended a preparatory visit before actual treatment visit for assessment purposes for suitability for sedation.^[7]^ The Scottish Dental Clinical Effectiveness Programme (SDCEP) in 2017 also recommended the use of introductory (acclimatization) visit.^[2]^

Although authors suggested/recommended the use of an acclimatization visit, to our knowledge the existing literature does not provide any information about the efficacy of the use of an acclimatization visit of nitrous oxide sedation on the acceptance of the pediatric patients for the procedure and whether this introductory visit would result in a reduction in the stress level and an improvement of the patients’ behavior during the procedure.

Measuring dental anxiety through clinical observation is of limited value as it relies on subjective assessment.^[8]^ The lack of standardized dental anxiety measuring technique highlights the immense need for an objective assessment that will overcome the human bias encountered with the routinely used subjective assessments. A considerable amount of literature has been published on saliva as a noninvasive biological biomarker of stress.^[9]^ One of the major enzymes of saliva is salivary alpha amylase (SAA). Synthesized and secreted by acinar cells of the salivary glands, mainly the parotid gland.^[10]^ Several studies have reported the correlation between dental anxiety and the increase in SAA and cortisol levels.^[11]^ The present study set out to assess the values of SAA and salivary cortisol levels as measures of anxiety in children to compare the effect of an acclimatization visit of IHS before dental treatment with those treated routinely without an acclimatization visit.

### 1.1 Study aim

This single-center, single blinded, parallel group randomized controlled two arm superiority trial aims to study the effect of a 15 minutes nitrous oxide IHS acclimatization visit on the behavior and anxiety levels of children going through dental treatment as measured by the SAA and Cortisol levels and to compare this effect with the children who go through dental treatment under IHS without the acclimatization visit.

### 1.2 Research hypothesis

The utilization of an acclimatization visit of nitrous oxide sedation before commencement of treatment will result in improvement in child's behavior and reduction in the anxiety levels during dental treatment as measured by the salivary Amylase and Cortisol levels.

## 2 Methods

The proposed study is a single-center, single blinded (to the dentist providing dental treatment), parallel group randomized controlled two arm superiority trial, adhering to the guidelines of the SPIRIT 2013 ^[12]^

Proposed start date: September 1st, 2019
Proposed end date: February 28th, 2020

### 2.1 Primary outcome

Improvement in behavior and anxiety levels. The primary outcome measured by:

(1) Physiologically by measuring anxiety-related changes using salivary levels of Alpha Amylase and Cortisol.
(2) Anxiety scores at baseline (initial visit) and after treatment (second visit) using a component from the indicator of sedation need (IOSN).
(3) Behavior score will be recorded using, Frankl behavior rating scale (FBRS).

### 2.2 Secondary outcomes

The following significant outcomes will be classified as evidence of beneficial effect of the acclimatization IHS visit: satisfactory completion of the required dental treatment and children's and parents’ acceptance of dental treatment with or without acclimatization visit. The secondary outcomes are measured by:

(1) Guardian/child quantitative questionnaire.
(2) Records from clinical notes

All the parents of children aged 5 to 15 years referred for pediatric specialist treatment in Dubai Dental Hospital in need of treatment under IHS within 6 months’ period will be invited to participate in the study. Inclusion criteria will include: healthy children with American Society of Anesthesiologists (ASA) classification of I or II aged 5 to 15 years in need of dental treatment under IHS, with no learning disabilities and suitable for nitrous oxide/oxygen IHS. UAE and non-UAE nationals’ parents and children will be eligible to participate in the study. Included children should have no previous experience of nitrous IHS. Exclusion criteria will include children with special healthcare needs and/or medically compromised, as well as those with complex medical history (ASA III, ASA IV) or diagnosed with a psychiatric disorder, children who lack communication due to language barrier. Participants will also be excluded if their parents refuse to sign the consent.

**Figure 1:**
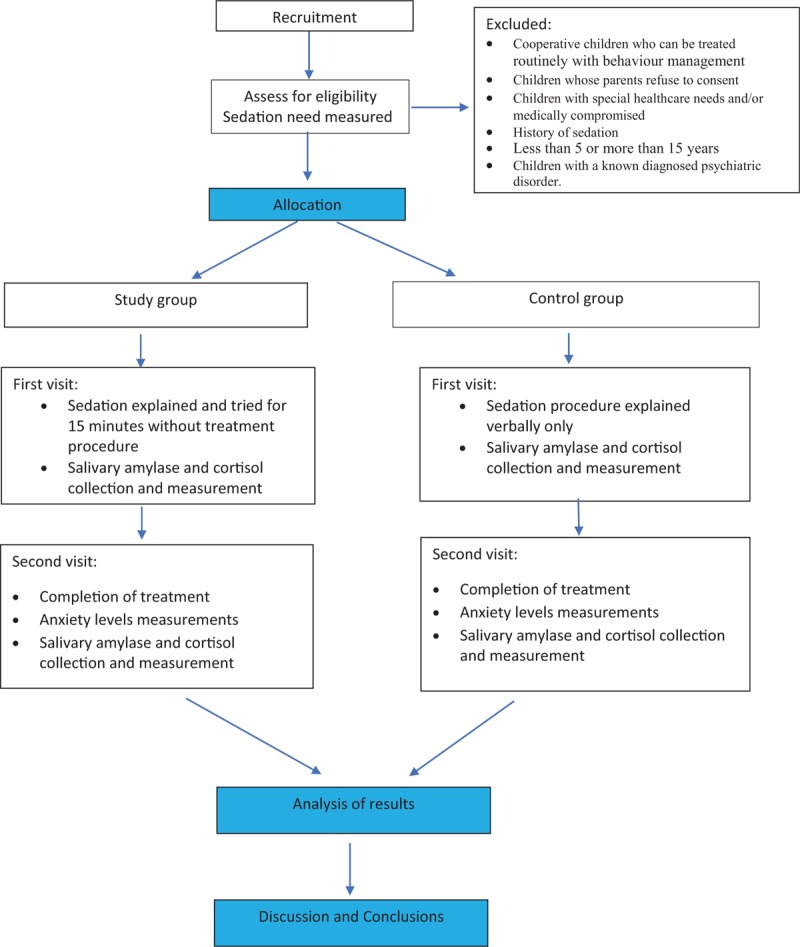
Flowchart of the study design.

A flowchart of the study design is presented in Figure 1.

After baseline examination by the clinical team, eligibility verification, the primary investigator will obtain the participant's assent and parents’/guardian's consent submission (Appendix I, http://links.lww.com/MD/D198), participants will be randomized to either of the two study groups (30 patients per group). The study group will include families (children and parents) who would attend a visit for prevention where IHS will be introduced and tried while the control group will be the families who would attend for a prevention visit and discussion of IHS procedure only.

Block randomization with a 1:1 allocation will be performed. Concealment of allocation will be achieved by using identical, sealed, sequentially numbered, opaque envelopes that will contain group assignment. The envelopes will be opened sequentially by a dental assistant, only after the envelope has been irreversibly assigned to each participant and will remain unknown to the clinical team.

At the initial acclimatization visit, the sedation need score will be recorded using the pediatric indicator of sedation need (p-IOSN). Acclimatization visit will be carried out by a single pediatric dentist who will not be involved in the treatment of the patients. To be consistent, an information script will be prepared and rehearsed so that the same instructions about the IHS will be delivered to all participants. During the acclimatization visit, the pediatric dentist will record the p-IOSN score based on the answers of the participants to the components of the index. At the beginning of the visit, an unstimulated salivary sample of 1.5 to 2 ml will be collected to measure the levels of Alpha Amylase and Cortisol. For the intervention group only, a 15 minutes nitrous oxide IHS will be administered without any dental procedures performed.

During the second visit, two unstimulated 1.5 to 2 ml samples of saliva will be collected 15 minutes before the start of the procedure and 15 minutes after the conclusion of the procedure. The dental treatment for both study and control participants will include administering local analgesia and restoring decayed teeth with or without pulp therapy and/or dental extractions. The intervention will be carried out by a single pediatric dentist.

**Figure 2:**
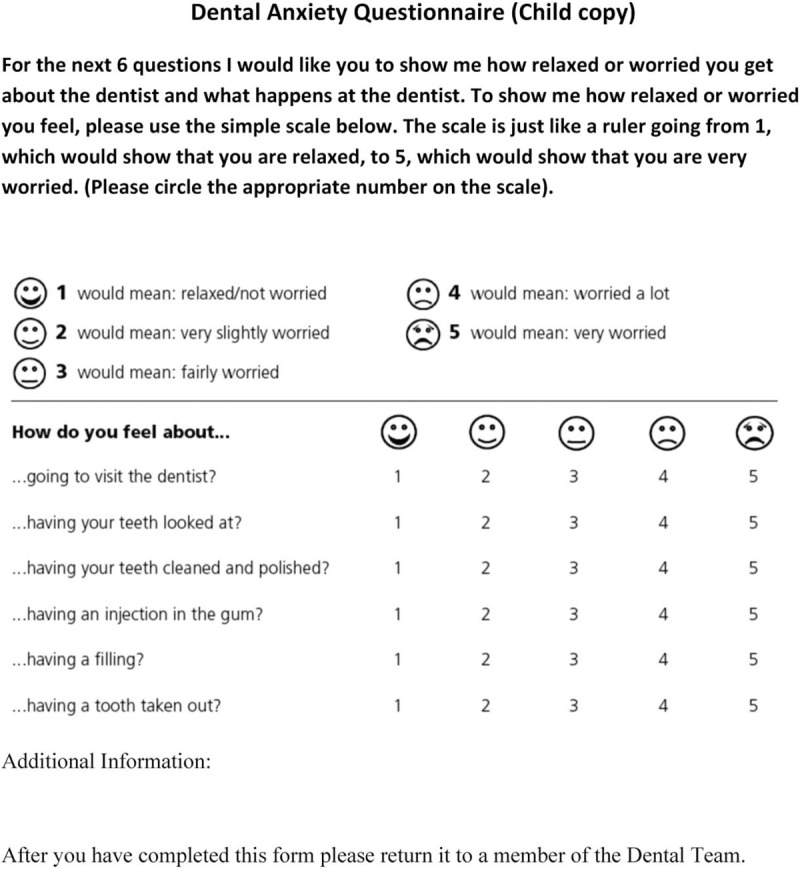
Modified child dental anxiety scale (Source: SDCEP Oral Health Assessment and Review 2017).

The 3 components of p-IOSN (Fig. 2, Tables 1 and 2) include:

(1) the modified child dental anxiety scale (MCDASf) (Fig. 2) using the SDCEP recommended shortened MCDASf questionnaire without the last two questions (on dental sedation and general anesthesia) for all anxious children.^[13]^ The MCDASf has been used widely in many dental behavioral studies.^[14–16]^
(2) the treatment complexity rank score (Table 1) records the medical status which is based on the patient's (ASA) classification (Table 2).

**Table 1:**
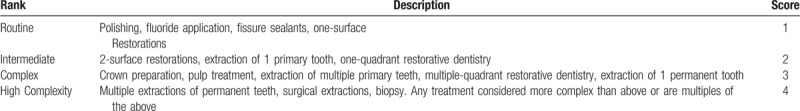
Treatment complexity rank score for the pediatric version of the indicator of sedation need (p-IOSN).

**Table 2:**
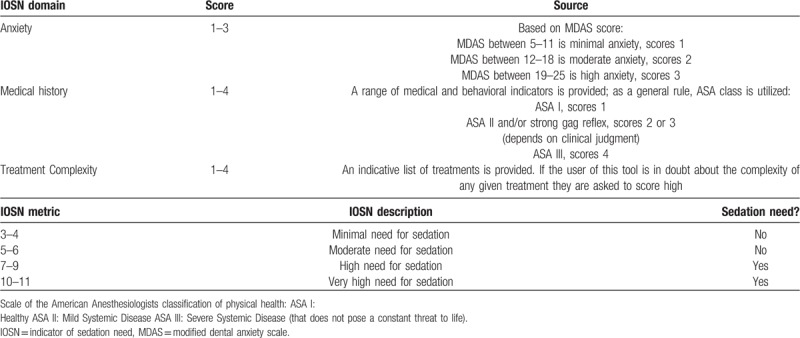
Summary of p-IOSN scoring system modified for children.

Madouh and Tahmassebi^[17]^ validated a pediatric version (p-IOSN). They used two anxiety questionnaires; The facial image scale was used for children under 10 years of age and the faces version of the MCDASf for older children. They also modified the last component of the IOSN which is the “treatment complexity” in order that it would be applicable to children's treatment rather than adults. Each question has five faces ranging from a very happy face to a very sad face and score range of 1 to 5. The total score range is 6 to 30. Children with a score ≥19 will be considered to have severe dental anxiety while those with a score of <19 will be considered to have no to moderate anxiety.

The MCDASf questionnaire will be administered in English. However, Arabic language translated versions will be used when required. The English version was translated into the Arabic language using the forward and backward translation method. The translation was checked by an independent bilingual expert who resolved concerns and discrepancies. To make sure that the translation was effective a back-translation to the English language was done by an independent translator who back-translated the questionnaire and discrepancies in the translation version were resolved.

In addition to the p-IOSN, physiological anxiety related changes will be assessed by measuring the levels of SAA and Cortisol. Salivary samples of 1.5 to 2 ml unstimulated saliva will be collected and analyzed using Expanded Range High Sensitivity Salivary Amylase and Cortisol Enzyme Immunoassay Kit (Bio Medical Scientific Services LLC ISO 9001:2008 Registered Firm. Certificate No.: DQU-12422; Hili Industrial Area, Al Ain, UAE).

Further subjective assessment of behavior and anxiety (Table 3) will be recorded using the numerical FBRS scores (1 to 4 where 1 is extremely uncooperative).^[18]^ Parents and children will be requested to complete a short questionnaire at the end of the treatment visit to measure their acceptance/satisfaction of dental treatment with or without acclimatization visit (Tables 4 and 5).

**Table 3:**
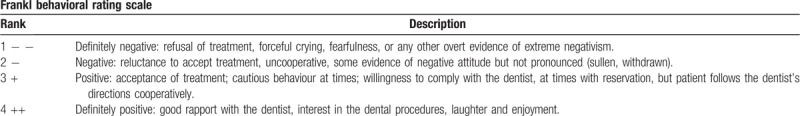
Frankl behavior rating scale.

**Table 4:**
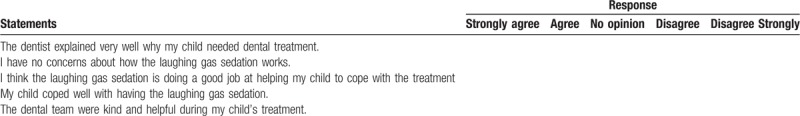
Parent's dental treatment acceptance questionnaire.

**Table 5:**
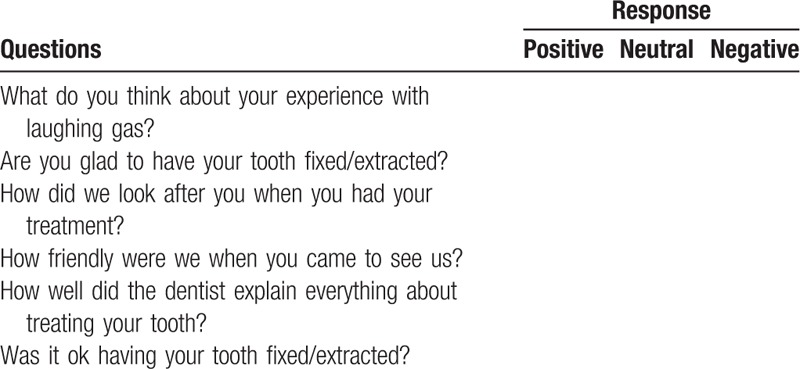
Children's dental treatment acceptance questionnaire.

### 2.3 Sample size calculation

The size of the sample necessary to detect a statistically significant difference was determined with a power calculation based on the results from studies carried out using the same outcome measures to evaluate behavior.^[19]^ To achieve an 80% power to detect a difference between the two groups a sample size of 30 patients was required. To compensate for dropout rate of 20% to 30%, that is, we decide to recruit 40 children in each group.

### 2.4 Patient and public involvement

#### 2.4.1 Development of the research question and outcome measures informed by patients’ priorities, experience, and preferences

A survey on the preference for the use of a familiarization to IHS before commencement of treatment was carried out involving 15 children, aged 5–15 years. The results were inconclusive with eight out of 15 children expressing preference for starting treatment without familiarization to sedation. This formed our primary outcome measure on comparing changes in self-reported anxiety scale in children who have familiarization visit and those who do not.

#### 2.4.2 Involvement of patients in the design of this study

As part of the above survey, we also asked children how they would feel completing MCDASf (anxiety form) and, we explained to them and demonstrated the process of collecting the salivary sample. 12 out 15 children were happy with the salivary sample collection method, three children found it uncomfortable. All the children in this survey mentioned that completing MCDASf was fine.

#### 2.4.3 Involvement of patients in the recruitment to and conduct of the study

The patients who will be recruited in this study will already be indicated for treatment under IHS in any case.

#### 2.4.4 Study results dissemination

We will have a dissemination event inviting participants/guardians, dental teams including general dental practitioners, dental nurses, specialists, and consultant to discuss our findings as it will be of interest to all those who manage children with dental anxiety. The results of the study will be presented at national and international conferences and will be submitted to a high impact factor dental journal.

#### 2.4.5 Assessment of the burden of the intervention the participants

We discussed the randomization with the children in the survey and 10 out of 15 children were happy to be randomized and were not overly concerned with whichever arm they would be randomized to. For children younger than 10 years, the opinion of the parents/guardians will be sought.

### 2.5 Ethical considerations and dissemination

All parents/caregivers and children fulfilling the inclusion criteria will be eligible for participation. In addition, to a routine verbal and written information and treatment consent form signed by parent/caregiver of the child before treatment under IHS, participating children's parents/caregivers will be informed verbally and in writing about the study. Participants and their parents/guardians will be appropriately informed about the objectives of the study; each participant will be ensured anonymity. Participants and their legal guardians will be informed about their right to withdraw from the study at any point. Appendix 1, http://links.lww.com/MD/D198 includes the consent forms both in English and Arabic. Following data analysis, collective results will be published. A signed, informed consent form will be required for participation from the parents/caregivers. The study has been approved by the ethics and research committee of Mohammed Bin Rashid University of Medicine and Health Sciences (Reference: MBRU-IRB-2018-014).

The results of the study will be disseminated through peer-reviewed publication/s, conference presentations, and the University web site. We will also have a dissemination event inviting dental teams including general dental practitioners, dental nurses, specialists, and consultant to discuss our findings as it will be of interest to all those who manage children with dental anxiety. Alongside these traditional outputs, our Forum, will help produce a simple and short end of trial English summary. This will be outreached to the wider community.

### 2.6 Data management and statement

Each participant will be assigned a unique study code number. Participant identifiers which will be collected will include name, phone number and clinic file number. Clinical recording sheets, Alpha Amylase and Cortisol immunoassay data will only be labelled using the code number.

### 2.7 Data management committee

This will comprise of the PI Mawlood Kowash, administrative staff and the study biostatistician. This team will be responsible for data entry and verification as well as ensuring that the collected data is stored securely. The list connecting the participant name/contact details with the study code number will be kept in a locked cabinet in the principal investigator's office. Only the principal investigator will have access to this list.

Anonymous participants’ data may be shared on request to promote culture of openness and an increased sharing of research data. Our information sheet and informed consent clearly state that the collected data are intended to be used for research purposes, and possibly published in dental journals and presented at conferences. All the anonymously collected data can be shared upon request from the primary investigator once the study is completed. The data will be stored for five years after the final publication to be shared upon request.

### 2.8 Statistical analysis

Data analysis will be conducted, after investigating distribution normality, with the appropriate parametric or non-parametric statistical tests using the statistical software SPSS 24.0.0 (^©^SPSS Inc, Chicago, Ill). The level of significance will be set at *P* < .05. Use of Descriptive statistics and quantitative data will be analyzed using means and standard deviations if normally distributed or medians and interquartile range if skewed. Mean changes of anxiety scores and corresponding 95% confidence intervals will be determined to compare the two treatments (sedation with familiarization and sedation without familiarization). *T*-tests will be used to compare quantitative data if normally distributed or Mann–Whitney *U*-test if skewed. The Chi-square test will be used to investigate association of categorical data. Box plots, individual and mean profiles of anxiety scores at each assessment time point for each treatment will be produced.

## 3 Discussion

This study/protocol aims at providing evidence-based answers for an aspect of IHS that has been left open to practical interpretation over the years; namely the merits of the use of an acclimatization visit before nitrous oxide IHS treatment session.

IHS with nitrous oxide has been at the helm of sedation in dentistry for more than 170 years, but recently there has been a major call for more robust evidence to back its use generally and in dental practice.^[17,20]^ Nevertheless, IHS use has contemporarily been strongly recommended by major organizations that champion the dental welfare of children globally. For example, the American Academy of Pediatric Dentistry (AAPD) recognizes nitrous oxide/oxygen inhalation as a safe and effective technique to reduce anxiety, produce analgesia, and enhance effective communication between a patient and health care provider (AAPD 2015).^[1]^ The SDCEP (2017) recommended the use IHS with nitrous oxide/oxygen as the preferred technique for conscious sedation, unless judged to be unsuitable for the patient and clinical need and that IHS with nitrous oxide/oxygen is the only standard technique for children.^[2]^ These endorsements echoed other major dental guideline issuers such as the UK dental faculties of the Royal Colleges of Surgeons and the Royal College of Anaesthetists. The latter, in 2015, recommended two visits for IHS - one preparatory (for assessment purposes for suitability for sedation only) and the other one for actual treatment.^[7]^

Authors of major textbooks in pediatric dentistry^[6]^ had suggested since the 1980's the utilization of an acclimatization visit before dental treatment under nitrous oxide IHS, thus advocating such practice under the well-known pediatric dentistry principle of Tell-Show-Do.^[21]^ However, there remains a paucity of data and controversy regarding the specific use of acclimatization visit for dental sedation treatment to help reduce anxiety and enhance the acceptance of dental treatment and cooperation of child dental patients.

The only resource that recommended the use of introductory (acclimatization) visit was the SDCEP in 2017.^[2]^ They issued the following statement: “*A brief trial of nitrous oxide/oxygen at the assessment appointment may be helpful for the psychological preparation of some children*.” However, specific evidence to support this statement was unclear. Therefore, at the present time, the recommendations are based on expert opinions and are not evidence-based.

The findings of this unique research measuring the difference in the physiologic effect of the acclimatization visit as determined by the levels of SAA and Cortisol will hope to provide some evidence for the guidelines on whether to have a separate session for acclimatization for children requiring dental treatment under IHS. Such an intervention, if tested and approved to be successful and effective in changing the physiological parameters, will increase the efficacy of nitrous oxide IHS to treat anxious children in the clinic and avoid the need for dental GA, an option with significant morbidity and is not without the risk of mortality.^[22]^ Furthermore, the discomfort produced and the inconvenience of a prolonged time of no oral feeding make dental GA a no longer recommended “best practice” for dental care.^[23–25]^ In addition, dental treatment under conscious sedation will reduce dental treatment cost by about a third by avoiding expensive GA hospital admissions.^[26]^

## Acknowledgments

The authors would like to acknowledge Dr Amar Hasan for his contribution in the statistical design of the protocol.

## Author contributions

**Conceptualization:** Mawlood Kowash, Manal Al Halabi, Rawan Awad, Najla Alderei.

**Data curation:** Mawlood Kowash.

**Formal analysis:** Mawlood Kowash, Manal Al Halabi, Anas Salami, Rawan Awad, Najla Alderei, Iyad Hussein.

**Funding acquisition:** Mawlood Kowash, Manal Al Halabi.

**Investigation:** Rawan Awad, Najla Alderei.

**Methodology:** Mawlood Kowash, Manal Al Halabi, Anas Salami, Rawan Awad, Najla Alderei, Ahtiq Wahab, Iyad Hussein.

**Project administration:** Mawlood Kowash, Manal Al Halabi.

**Resources:** Mawlood Kowash.

**Supervision:** Mawlood Kowash, Manal Al Halabi.

**Validation:** Mawlood Kowash.

**Visualization:** Manal Al Halabi.

**Writing – original draft:** Mawlood Kowash, Manal Al Halabi.

**Writing – review and editing:** Mawlood Kowash, Manal Al Halabi, Anas Salami, Rawan Awad, Najla Alderei, Iyad Hussein.

Mawlood Kowash orcid: 0000-0002-4721-3789.

## Supplementary Material

Supplemental Digital Content
