# Supplementary material for: A study protocol of a single-center investigator-blinded randomized parallel group study to investigate the effect of an acclimatization visit on children's behavior during inhalational sedation in a United Arab Emirates pediatric dentistry postgraduate setting as measured by the levels of salivary Alpha Amylase and Cortisol
Source: Medicine (Baltimore). 2019 Aug 30;98(35):e16978. doi: 10.1097/MD.0000000000016978 (PMC6736477; doi:10.1097/MD.0000000000016978)
Supplement: Supplemental Digital Content [file medi-98-e16978-s001.docx]

**Informed Consent to Participate in a Research Study**

**This study has been approved by the MBRU-Institutional Review Board [IRB Approval # MBRU-IRB-2018-014]**

**Principal Investigator:** Najla Alderei, Rawan Awad

**Address:** Hamdan Bin Mohammed College of Dental Medicine, Mohammad Bin Rashid University of Medicine and Health Sciences, Building No 34, Dubai Health Care City

Dubai - UAE

**Phone: 04-383 8909**

**Site where the study will be conducted:** Dubai Dental Hospital, Hamdan Bin Mohammed College of Dental Medicine

**Research Title:** Effect of nitrous oxide inhalation sedation acclimatization visit on children’s anxiety levels as measured by salivary amylase and salivary cortisol levels

You and your child are invited to participate in this research study conducted at Dubai Dental Hospital, Hamdan Bin Mohammed College of Dental Medicine, Mohammad Bin Rashid University of Medicine and Health Sciences (MBRU). Please, take your time to read the following information carefully, before you decide whether you wish to take part in this research study or not. You are encouraged to ask the study investigator if you need any additional information or clarification about what is stated in this form and/or in the research study as a whole. You are also free to take this information sheet and consult with your doctor or other health professionals. Please note that, should you decide to participate, you are free to withdraw at any time without any consequence.

1. **Purpose of the Research Study and Overview of Participation**

| Laughing gas (inhalation sedation) is a proven safe method for reducing children’s dental anxiety and is used worldwide for decades. Your child requires laughing gas for his/her dental treatment.  The purpose of this study is to help dentists and dental care providers to identify whether or not a laughing gas introductory/familiarization visit is effective in making anxious children more relaxed and accepting of dental treatment. Your child will be randomly assigned to a first visit which will either be: **1) Group “A”** where you and your child would attend a visit for tooth brushing advice (no treatment) and laughing gas will be used and introduced to familiarize your child or **2) Group “B”** where parents/children would attend for tooth brushing advice (no treatment) and a discussion of inhalation sedation only would take place without actual usage of laughing gas. To record anxiety related changes, three saliva samples will be collected at three different occasions: before the first visit, before, and after the second visit dental procedure. |
| --- |

1. **Any Risks as a Result of Participating in the Study**

| Possible risks: Nausea and vomiting in 1-10% of cases and Occasional headache  You and your child have been chosen because your child is referred/in need of dental treatment under laughing gas sedation. The results of this study are intended to be used for research purpose, and possibly published in Dental Journals and presented at conferences. **There will be no mention of specific individuals**. |
| --- |

1. **Any Benefits as a Result of Participating in the Study**

| There may be no direct benefit to your child, however we hope to understand more about how you and your child feels about the dental treatment using laughing gas sedation and by taking part we can find out if an inhalation sedation (laughing gas) introductory or familiarization visit is effective in making children more relaxed and accept dental treatment. Please note that the cost of the visit will be covered by a research grant because many insurance companies do not cover the cost of the introductory or familiarization visit. |
| --- |

1. **Any Alternative Treatment**

| No, as laughing gas treatment is indicated for your child anyway. |
| --- |

If you agree to take part in this research study, please, be ensured that the obtained information will be kept confidential. Unless required by law, only the study investigator or designee, the MBRU-Institutional Review Board (MBRU-IRB), and/or inspectors from governmental agencies will have direct access to your information.

In case of any adverse event, as a result of the study, there will be no compensation to cover such expenses if it is not covered by a third party or governmental insurance.

**Investigator’s Statement:**

I have reviewed, in detail, the informed consent document for this research study with (name of patient, legal representative, or parent/guardian) the purpose of the study and its risks and benefits. I have answered to all the participant’s questions clearly. I will inform the participant in case of any changes to the research study. The participant will be free to withdraw this consent and discontinue participation, however unless she/he asks us not to, the information already collected shall be used in the analysis.

Najla Alderei, Rawan Awad

**Name of Investigator or Designee Signature Date & Time**

**Patient’s Participation:**

I have read and understood all aspects of the research study and all my questions have been answered. I voluntarily agree to be a part of this research study and I know that I can contact **Dr. Najla Alderei, Dr Rawan Awad,** or any of his/her team involved in the study in case I have any questions. If I feel that my questions have not been answered, I can contact the MBRU-IRB. I understand that I am free to withdraw this consent and discontinue participation in this project at any time, even after signing this form, and it will not affect my care or benefits. I know that I will receive a copy of this signed informed consent.

__________________________ __________________ _______________

**Name of Patient/Legal Signature Date & Time**

**Representative or Parent/Guardian**

**Name of the Witness Witness’s Signature**

**(if patient, representative or parent do not read)**

**Date & Time**

**INFORMED CONSENT FORM (Arabic)**

**نموذج بيانات الوالدين والموافقة المسبقة**

**عنوان الدراسة:**

**التقييم الموضوعي لمستوى القلق لدى الأطفال و مدى جدوى الزيارة التعريفية**

**(التقييمية) للغاز الضاحك و أثرها على سلوك الطفل.**

لقد تم إختيار إبنك/ إبنتك للمشاركة في الدراسة نظرا لإحتياجه لجلسات المهدِي بإستخدام الغاز الضاحك لإتمام علاج أسنانه

(أسنانها)

**الباحث الرئيسي: د. نجلاء الدرعي و د. روان عواد**، قسم طب أسنان الأطفال، كلية حمدان بن محمد لطب الأسنان، مبنى رقم 34، مدينة دبي الطبية، دبي، الإمارات العربية المتحدة، هاتف رقم: 9004 593 (050) أو 043838909

يرجى مراجعة نموذج البيانات هذا بروية، ولا تترددوا في استشارة أو مناقشة هذه الدراسة مع طبيب الأسنان الذي تتعاملون معه، و/أو الزملاء و/أو الأسرة و/أو الأصدقاء و/ أو الطبيب قبل اتخاذ قرار بالمشاركة من عدمها. إذا كان لديكم أي أسئلة بخصوص هذه الدراسة أو أية مسائل تتعلق بها، فلا تترددوا في طرحها على الباحث الرئيسي الموضح أعلاه. قد يحتوي نموذج الموافقة هذا على كلمات يتعذر عليكم فهمها، ومن ثم يُرجى الاستفسار من مسئول البحث عن أي كلمات أو معلومات يتعذر عليكم فهمها بشكل واضح.

**الغرض من الدراسة**

تُجرى هذه الدراسة في كلية حمدان بن محمد لطب الأسنان بجامعة محمد بن راشد للطب و العلوم الصحية، قسم طب أسنان الأطفال لتقييم مستوى القلق لدى الأطفال و مدى جدوى الزيارة التعريفية (التقييمية) للغاز الضاحك و أثرها على سلوك **إجراءات الدراسة**

إذا اخترت المشاركة في هذه الدراسة، فلابد من اتخاذ الإجراءات التالية: سيُطلب منك ومن طفلك ملء استبيان في عيادة الأسنان بعد الفحص و العلاج وسيتم جمعها من قبل المساعد. (يرجى الرجوع إلى النسخة المرفقة).

سيتم إدراج إسم إبنك/ إبنتك عشوائيا في إحدى المجموعتين:

1. أنت و إبنك/ إبنتك تزورا العيادة و يقوم طبيب الأسنان بشرح الطريقة المثلى لتنظيف أسنان إبنك/ إبنتك ثمَ يقوم بشرح عملي لكيفية عمل الغاز الضَاحك و تجربته و لكن بدون علاج الأسنان في هذه الزيارة.
2. سيقوم طبيب الأسنان بشرح الطريقة المثلى لتنظيف أسنان إبنك/ إبنتك و شرح كيفية عمل الغاز الضَاحك فقط.

لن يتم حرمان طفلك من العلاج نتيجة لمشاركتكم في هذه الدراسة. ويمكنكم التوقف عن المشاركة في هذه الدراسة في أي وقت. ومع ذلك، إذا كنتم ترغبون في وقف المشاركة، يُفضل التحدث مع موظف البحث أولا.

**المخاطر والإزعاج**

العلاج بإستخدام مهدِي الغاز الضَاحك لا يؤدي في العادة لمخاطر معروفة أو إنزعاج معروف و لكن بعض الأطفال ممكن يشتكى من صداع أو تقيء (إستفراغ).

**الفوائد**

قد يعود على طفلك أو قد لا يعود عليه فوائد مباشرة من المشاركة في هذه الدراسة. ونأمل أن تساعد المعلومات التي نجمعها المهنة في توفير أفضل رعاية ممكنة لصحة الفم للمرضى و علاج التوتر و القلق عند الأطفال.

**التكلفة/ المدفوعات**

لن تتحمل أي تكلفة على المشاركة في الدراسة ولن تتلقى أية مدفوعات أو تعويضات عن أي نفقات تتعلق بالمشاركة في هذه الدراسة وبمعني آخر: لن يكون هناك أي التزام من جانبك تجاه المشاركة في الدراسة.

**السرية**

جميع المعلومات التي يتم الحصول عليها من هذه الدراسة سرية وستظل كذلك. وقد يتم نشر المعلومات التي يتم جمعها في هذه الدراسة أو تقديمها في المحافل العامة؛ ومع ذلك، لن يتم استخدام اسم طفلك وبيانات التعريف الأخرى الخاصة به أو الإفصاح عنها. وفي حال نشر تلك البيانات، سيتم حماية هويتك (وهوية طفلك) والتعامل معها على أنها سرية وفقا لقانون المعلومات الصحية الشخصية لدولة الإمارات العربية المتحدة. ولحماية هويتك، سيتم منح كل مشارك رقم دراسة بدلا من اسمه في جميع الوثائق ذات الصلة بالدراسة. وسيتم استخدام جميع المعلومات التي يتم الحصول عليها من هذه الدراسة بدقة لأغراض البحث فقط. وإذا تم استخدام معلومات الدراسة في أي بحث لاحق، سيتم الحصول على موافقتك.

وعلاوة على ذلك، قد يراجع مجلس أخلاقيات البحث بكلية حمدان بن محمد لطب الأسنان سجلات الدراسة لأغراض ضمان الجودة فقط. وعلى الرغم من الجهود المبذولة للحفاظ على سرية معلوماتك الشخصية، لا يمكننا ضمان السرية المطلقة، فقد يتم الإفصاح عن بياناتك الشخصية إذا اقتضى القانون ذلك.

كما ستبقى جميع السجلات المتعلقة بهذه الدراسة في منطقة آمنة ومغلفة ولن يُسمح بالوصول إلى تلك السجلات سوى للأشخاص المصرح لهم بذلك فقط. وإذا تطلب الأمر أن يتم تقديم نسخ من السجلات الطبية/ البحثية الخاصة بطفلك إلى أي من الجهات المذكورة أعلاه، سيتم إزالة اسمه/ اسمها وجميع المعلومات التعريفية الخاصة به/ بها. ولن تُغادر أي من المعلومات التي تكشف أي بيانات شخصية، مثل اسمك/ اسم طفلك أو العنوان أو رقم الهاتف، كلية حمدان بن محمد لطب الأسنان.

**المشاركة الطوعية/ الانسحاب من الدراسة**

يعد قرارك بالمشاركة والسماح لطفلك بالمشاركة في هذه الدراسة أمر طوعي، ولك حرية رفض منح الموافقة بشأن مشاركة طفلك في الدراسة أو الانسحاب منها في أي وقت. وإذا شعر فريق البحث أن في مصلحة طفلك أن يتم سحبه/ سحبها من الدراسة، سيقومون بإزالة بياناتك دون موافقتك.

وسنقوم بإخطارك بأي معلومات جديدة قد تؤثر على صحة طفلك أو رعايته أو الرغبة في البقاء في هذه الدراسة.

**الأسئلة**

يرجى عدم التردد في طرح أية أسئلة بشأن الدراسة أو أي شيء يتعلق بها يتطلب مزيدا من التوضيح. للاتصال بموظفي البحث بشأن طرح أي سؤال، يرجى الاتصال على:

د. مولود الكواش على الرقم 9004 593 (050) أو 043838909

لا تُوقع على نموذج الموافقة هذا إلا إذا أُتيحت لك الفرصة في طرح أسئلة وتلقيت أجوبة مرضية على جميع أسئلتك.

**بيان الموافقة**

قرأت نموذج الموافقة هذا وأتيحت لي الفرصة لمناقشة هذه الدراسة مع **د.** **مولود الكواش** و/أو موظفي البحث التابعين لها، وكانت الإجابة على أسئلتي بلغة أفهمها. وقد تم شرح جميع المخاطر والمنافع والتكاليف والبدائل الخاصة بهذه الدراسة بدقة، وأعتقد أنه لم يتم التأثير عليّ بصورة غير ملائمة من قبل أي عضو من أعضاء فريق البحث للمشاركة في الدراسة من خلال أي تصريحات صريحة أو ضمنية. كما لم تؤثر أي علاقة بيني أو بين طفلي وفريق البحث على قراري في المشاركة. وأعلم أنه سيتم إعطائي نسخة من نموذج الموافقة هذا بعد التوقيع عليه، كما أعلم أن مشاركتي ومشاركة الطفل في الدراسة أمر طوعي وأنني قد أختار سحب طفلي منها في أي وقت. ومن ثم فإنني أوافق على المشاركة في هذه الدراسة البحثية بمطلق حريتي وأوافق على أن يشارك طفلي في الدراسة البحثية أيضا.

كما أعلم أن أية معلومات تتعلق بهوية طفلي ستظل سرية، ولكن لا يمكن ضمان تلك السرية. وأوافق على أن يتم فحص أي من السجلات الخاصة بي التي تتعلق بهذه الدراسة من قبل مجلس أخلاقيات البحث بكلية حمدان بن محمد لطب الأسنان لأغراض ضمان الجودة.

وبتوقيعي على نموذج الموافقة هذا، فإنني لم أتنازل عن أي من الحقوق القانونية المكفولة لي أو لطفلي كمشارك في دراسة بحثية.

توقيع أحد الأبوين/ الوصي القانوني: ___________________

التاريخ: ____________________ (يوم/ شهر/ سنة)

اسم الوالد/ الوصي القانوني طباعةً: _______________________________

أقر أنا، الموقع أدناه، وأصادق على أن المعلومات الواردة في نموذج بيانات المشارك والموافقة قد تم شرحها بدقة لي، وأنه قد تم فهمها بوضوح من قبل المشارك أو ممثل المشارك المقبول من الناحية القانونية، وأنه قد تم منح الموافقة بالمشاركة في هذه الدراسة من قبل المشارك أو ممثل المشارك المقبول من الناحية القانونية بمطلق حريته.

توقيع الشاهد: ____________________________

التاريخ: ___________________ (يوم/ شهر/ سنة)

اسم الشاهد طباعةً: ______________________________________
